# Early Weaning Stress Induces Intestinal Microbiota Disturbance, Mucosal Barrier Dysfunction and Inflammation Response Activation in Pigeon Squabs

**DOI:** 10.3389/fmicb.2022.877866

**Published:** 2022-05-31

**Authors:** Qianqian Xu, Huafeng Jian, Wenyan Zhao, Jiankui Li, Xiaoting Zou, Xinyang Dong

**Affiliations:** Key Laboratory for Molecular Animal Nutrition of Ministry of Education, Key Laboratory of Animal Feed and Nutrition of Zhejiang Province, College of Animal Science, Zhejiang University (Zijingang Campus), Hangzhou, China

**Keywords:** barrier function, early weaning, intestine, microbiota, pigeon squab

## Abstract

Early weaning stress has been reported to impair intestinal health in mammals. Like mammals, weaning of the pigeon squab, an altricial bird, is associated with social, environmental and dietary stress. However, understanding of weaning stress on intestinal functions is very limited in altricial birds, especially in squabs. This study was aimed to evaluate the effects of early weaning stress on intestinal microbiota diversity, architecture, permeability, the first line defense mechanisms, mucosal barrier functions, and immune cell responses. A total of 192 newly hatched squabs were randomly allocated into two groups, one weaned on day 7 and the other remained with the parent pigeons. Mucosal tissue and digesta in ileum, as well as blood samples, were collected from squabs (*n* = 8) on days 1, 4, 7, 10, and 14 postweaning. Our results showed that weaning stress induced immediate and long-term deleterious effects on both growth performance and intestinal barrier functions of squabs. Early weaning significantly increased ileal bacterial diversity and alters the relative abundance of several bacteria taxa. Weaning stress can also cause morphological and functional changes in ileum, including an atrophy in villi, an increase in permeability, and a variation in the mRNA expression of genes encoding mucins, immunoglobulins, tight junction proteins, toll-like receptors, and cytokines, as well as the concentration of secretory IgA. We concluded that the impaired intestinal barrier functions accompanied with early weaning stress seems to be the main reason for the poor growth rate after weaning in squabs. In addition, the disturbance of intestinal microbiota of early weaning stress in squabs coincided with dysfunction of intestinal mucosal barrier and activation of inflammation cell responses that were possibly mediated *via* the activation of toll-like receptors.

## Introduction

The pigeon is an altricial bird, one of the few birds that can secrete nutrient solution (pigeon milk) to feed offspring (Sales and Janssens, 2003). The young pigeons in the stage from hatch to the natural weaning and leaving the nest are called squabs. As the main product of meat pigeon breeding, they have extremely high nutritional value and are known as “one pigeon is better than nine chickens” (Xu et al., 2020). Thus, the pigeon squab is an important representative of high-quality animal products and the consumer demand for squabs is increasing (Ji et al., 2020). However, the natural feeding mode by parent pigeons restricts the production efficiency of squabs, which has become a restrictive factor in the pursuit of maximum economic benefits. Under natural conditions, the squabs are completely dependent on their parents’ feeding, and usually wean on the days 18 ~ 28 post-hatch (Sales and Janssens, 2003). If the squabs weaned and left the nest earlier, the parent pigeons could lay their eggs 10–20 days in advance, and the annual production of each parent pigeon would increase from 6 to 10 ~ 15 pairs. Therefore, artificially feeding early weaned squabs can reduce the burden of breeding pigeons and shorten the breeding cycle, which is an important measure to improve the production efficiency of pigeon squabs. However, the early weaned squabs were inferior to those fed by parents in terms of growth performance and immunity (Wen et al., 2022).

Similar to early weaned mammals, early weaned squabs should undergo the process of changing from parent pigeon feeding to artificial feeding, and from a dependent environment where parents and young live together to an independent life. This will inevitably cause severe early life stress responses physically and psychologically in squabs like mammalian weaning stress (He et al., 2011). Early weaning stress in mammals such as piglets and young ruminants has been generally studied. Evidence emerged that early weaning stress could lead to intestinal dysfunction and atrophy, imbalance of intestinal microbiota, increased dyspepsia and diarrhea, and impaired mucosal barrier, which are responsible for the restrained growth of piglets (Smith et al., 2010; Hu et al., 2013; Chen et al., 2017). Similarly, weaning stress stunted gastrointestinal function especially ruminal development in weaned calves, consequently often leading to sickness and inferior calf performance (Hulbert et al., 2011; Zhou et al., 2012; Li et al., 2018). It can be concluded that the maintenance of normal intestinal functions is important for animal health and hence productivity. Therefore, we accordingly hypothesized that artificial feeding early weaned squabs would retard the squab growth by affecting development of the intestinal functions in squabs.

On the basis of previous studies on porcine and ruminant, understanding the intestinal injury caused by weaning stress is helpful for farms and researchers to find corresponding solutions to the problem (He et al., 2011; Wijtten et al., 2011; Malmuthuge et al., 2013; Ji et al., 2021). Unfortunately, the knowledge about the effect of early weaning on intestinal functions in altricial birds is scanty. Considering that understanding how stress affects the intestinal health of squabs could provide clues for subsequent mitigation of this impairment, the present study is designed to clarify the specific process of injury in intestinal functions by analyzing its morphological and barrier functional changes, as well as its ecological niche shifts, during the development of early weaned squabs. This is the first attempt to study the intestinal microbiota and barrier function in altricial birds under early life stress, and our results would provide potential new directions for exploring the mechanism of weaning stress on the intestinal host–microbe interactions and means to improve the functions of intestine in weaned squabs and their retarded growth performance, thus increasing economic returns.

## Materials and Methods

All experimental protocols involving animals were approved by the Animal Care and Welfare Committee of Animal Science College and the Scientific Ethical Committee of Zhejiang University (No. ZJU2013105002; Hangzhou, China).

### Birds and Experimental Design

A total of 192 newly hatched squabs with similar body weight (BW) were selected on the day of hatch. They were pair-matched and allocated into the nests of parent pigeons to replace the fake eggs which were used to meet parent pigeons’ brooding characteristics. Each parent pair adopted two newly hatched squabs. These squabs were fed with pigeon milk by parent pigeons in a beak-to-beak manner. On the days 7 post-hatch, these squabs were divided into two groups randomly: the control group (CON) and the early weaning group (EW). Each group consisted of eight replications, and each replication included 12 squabs. The squabs in CON group continued to be fed and nursed by their parents, whereas the squabs in EW group began to separate from their parents and were fed with artificial pigeon milk (17.77% protein and 13.04 MJ/kg energy content). The environmental conditions of the two groups remained consistent. The ambient temperature was 18–26°C, and the relative humidity was 60–70%. The photoperiod was 12 h light–12 h dark throughout the total study period.

### Sample Collection

On the days of 1 (D1), 4 (D4), 7 (D7), 10 (D10) and 14 (D14) postweaning, eight squabs (one squab from each replication) were selected from the CON group and the EW group randomly for sampling, respectively. The squabs were fasted overnight (12 h) before weighing and slaughtering. Blood samples were collected from wing vein for determining serum endotoxin, diamine oxidase (DAO), and D-lactate. The selected squabs were all killed by cervical dislocation (squabs with body weight over 250 g were sedated before cervical dislocation). Ileal contents were collected to determine the microbiota diversity; ileal mucosa was collected to assess mRNA expression of genes related to mucosal barrier functions and concentration of secretory IgA (SIgA); ileal sections were collected for morphology analysis.

### Growth Performance

The BW of squabs was recorded as a replication basis on D1, D4, D7, D10, and D14 postweaning, respectively. Average daily gain (ADG) were calculated by period and cumulatively.

### 16S rDNA High-Throughput Sequencing

Total genome DNA from samples was extracted using CTAB/SDS method. DNA concentration and purity were monitored on 1% agarose gels. According to the concentration, DNA was diluted to 1 ng/μl using sterile water. The V3–V4 hypervariable region of the 16S rRNA genes was amplified by specific degenerate primer (341F-806R). All PCR reactions were carried out in 30 μl reactions with 15 μl of Phusion^®^High-Fidelity PCR Master Mix (New England Biolabs, Inc., Beijing, China), 0.2 μM of forward and reverse primers, and about 10 ng template DNA. Thermal cycling consisted of initial denaturation at 98°C for 1 min, followed by 30 cycles of denaturation at 98°C for 10 s, annealing at 50°C for 30 s, and elongation at 72°C for 60 s, and finally 72°C for 5 min. Mix same volume of 1× loading buffer (contained SYBR green) with PCR products and operate electrophoresis on 2% agarose gel for detection. Samples with bright main strip between 400 and 450 bp were chosen for further experiments. PCR products were mixed in equidensity ratios. Then, mixture of PCR products was purified with AxyPrepDNA Gel Extraction Kit (Axygen Scientific, Inc., Union City, CA, United States). The library quality was assessed on the Qubit@ 2.0 Fluorometer (Thermo Fisher Scientific, Inc., Waltham, MA, United States) and Agilent Bioanalyzer 2,100 system. At last, the library was sequenced on an Illumina Miseq/HiSeq2500 platform (Illumina, San Diego, CA, United States) and 250/300 bp paired-end reads were generated.

### Microbiome Analysis

Paired-end reads from the original DNA fragments were merged using FLASH which was designed to merge paired-end reads when at least some of the reads overlap the read generated from the opposite end of the same DNA fragment. Paired-end reads were assigned to each sample according to the unique barcodes. Sequences analysis were performed by UPARSE software package using the UPARSE-OTU and UPARSE-OTUref algorithms. In-house Perl scripts were used to analyze alpha (within samples) and beta (among samples) diversity. Sequences with ≥ 97% similarity were assigned to the same OTUs. We pick a representative sequence for each OTU and use the RDP classifier to annotate taxonomic information for each representative sequence. In order to compute Alpha Diversity, we rarify the OTU table and calculate two metrics: Chao 1 estimates the species richness and Shannon index estimates the species diversity. Rarefaction curves were generated based on Chao 1 estimator of species richness with 20 sampling repetitions at each sampling depth. Cluster analysis was preceded by nonmetric multidimensional scaling (NMDS) analysis based on the Bray–Curtis dissimilarity distance matrices, which was applied to reduce the dimension of the original variables using the MicrobiomeAnalyst platform. To identify differences of microbial communities between the CON and EW groups, analysis of similarities (ANOSIM) *post hoc* test based on the abundance of OTUs in samples were performed.

### Intestinal Morphology Under Optical Microscope

Approximately 0.5-cm ileal samples were collected and fixed in 10% neutral-buffered formalin solution. Each sample was dehydrated, cleared and embedded in paraffin. Serial sections (5 μm) were placed on glass slides and submitted to hematoxylin–eosin staining. Three staining images per sample (eight sample per group) were used to identify villus height, villus surface area, crypt depth, and the ratio of villi to crypt (VCR). The examination was performed with optical microscope (Nikon Corp., Tokoyo, Japan) using Image-Pro Plus 6.0 (Media Cybernetics, Inc., Rockville, MD, United States). At least 18 villi and crypts were measured per sample. The villus height and crypt depth were measured using the line tool of software, and the villus surface area was measured using the area tool of software.

### Secretory IgA Analysis

The homogenates of ileal mucosa were prepared with PBS for SIgA analysis. The SIgA concentration was determined with a commercial ELISA kit (Shanghai Enzyme-linked Biotechnology Co., Ltd., Shanghai, China). The standard samples and diluent solution were added at 100 μl per well in duplicate. The plate was incubated for 2 h at 37°C, and washed by washing solution for three times. The biotin-antibody was added at 100 μl per well, with incubation at 37°C for 1 h and washing for five times. The HRP conjugate was added at 100 μl per well, with incubation at 37°C for 1 h and washing for five times. Next, 100 μl color solution was added into each well and develop color at 37°C for 15 min. Finally, 50 μl stop solution was added into each well to stop the reaction. The absorbance changes at a wavelength of 450 nm were immediately determined with a SpectraMax M5 microplate reader (Molecular Devices, Sunnyvale, CA, United States). The final SIgA concentration was expressed as ng per mg of protein.

### Intestinal Mucosal Permeability

The levels of serum endotoxin, DAO, and D-lactate in serum were determined by a SpectraMax M5 microplate reader (Molecular Devices, Sunnyvale, CA, United States) using commercial ELISA kits (Shanghai Enzyme-linked Biotechnology Co., Ltd., Shanghai, China) according to the manufacturer’s protocol.

### RNA Extraction and Quantitative PCR Analysis

Total RNA of the ileal mucosa was extracted using the MolPure^®^ Cell/Tissue Total RNA Kit (Yeasen Biotech Co., Ltd., Shanghai, China) according to the instructions of the manufacturer. The extracted RNA was quantified by a NanoDrop ND-2000 spectrophotometer (NanoDrop Technologies Inc., Wilmington, DE, United States). The RNA integrity was verified by native RNA electrophoresis on a 1.0% agarose gel. Complementary DNA was synthesized from 1 μg of total RNA by Hifair^®^ III 1st Strand cDNA Synthesis SuperMix for qPCR (gDNA digester plus; Yeasen Biotech Co., Ltd., Shanghai, China) following the protocol of the manufacturer. The abundance of mRNA was assayed on the Bio-Rad CFX Manager 3.0 system (CFX96 Touch, Bio-Rad Laboratories, Inc., Hercules, CA, United States). The specific primers used are shown in Table 1. Hieff^®^ qPCR SYBR Green Master Mix (No Rox; Yeasen Biotech Co., Ltd., Shanghai, China) was used for PCR, consisting of an initial DNA denaturation of 95°C for 5 min, followed by 40 cycles of 95°C for 10 s and 60°C for 30 s. Glyceraldehyde-3-phosphate dehydrogenase was considered an appropriate endogenous reference. The average gene expression relative to the endogenous reference for each sample was calculated according to the 2^−ΔΔCt^ method (Schmittgen, 2001). The calibrator for each gene in the study was the average ΔCt value of the CON group.

**Table 1:** Gene-specific primers used for quantitative real-time PCR analysis.

Gene	Accession	Primer sequences (5'–3')	Product size (bp)
*CLDN1*	XM_005513213.2	TCTTTGGTGGGGTGATGT	109
		TTGACAGGGGTGTAAGGG	
*CLDN2*	XM_021283269.1	GTGCAGATGGGAACAAGGT	119
		GAGCCAAGGAAGCTACGG	
*CLDN3*	XM_005515008.2	ACCTCATCCCCGTCTCCT	109
		CAGCCCACGTAGAGCGA	
*CLDN4*	XM_021282730	AATGAAGCTGTTGGAAGGG	135
		ATCTGGGTTCAAAGATGGC	
*CLDN16*	XM_005513212.2	ACAAAATGCCGTGGACTGTG	134
		GCTGTGATCATCATGGCTCG	
*IL1-β*	NM_001282824.1	CTGGACTTGGAAGCGACA	102
		GGCCACCATGAGGACAG	
*IL4*	XM_005512458	CTGTGACAAGATGAACGCGA	123
		ATTCCTTCGAGGTGGTTGTGG	
*IL6*	XM_013369893.2	GTGCAAAGCTCAAGTCCAA	108
		AATAGCGAACAGCCCTCAC	
*IL10*	XM_021298374.1	GACCAGCACCCTTCACC	111
		TTGCTCCTCTTCTCGCA	
*IL12*	XM_021288847.1	GAGTGCCAGAAAGAAAAC	154
		CATACTGACATTGGGGTG	
*MUC2*	XM_021283472.1	GAATGTGCCAAACAAAGC	231
		CTGATGTACGCAAACCCT	
*MUC5AC*	XM_021299062.1	AAGACCTTTGACGGAGAC	136
		GGTTGATTGTTGGAGCAT	
*MUC13*	XM_005505089.3	TGGGATGTTCTTTCTTGT	246
		AGTGACTGCTATGGCTAA	
*OCLN*	XM_005509325.2	CAGGACGTGGCAGAGGA	105
		GTGGAAGAGCTTGTTGCGT	
*PIGR*	XM_005510390.3	GAGGGCTCTCCCACAAAGTC	136
		CTCACGCTCTCGTTGTCCTT	
*TJP1*	XM_021299314.1	GAACCAAAGCCAGTGTATG	159
		GGTCCCCTTCCTCTAATC	
*TJP2*	XM_021283254.1	CGTCCTCGGTCGTGTTCATC	228
		TGTAGTGAAACGACCAAGGAAGG	
*TLR2t1*	XM_021285395	GCTGGGACCCTCTTTCTTGG	156
		TCCTGAAAGCAGAGCCTTGG	
*TLR2t2*	XM_013372151.2	CTACCTGGAGTGGCCTCCT	137
		ACATGCAAGGCAAACAAATCC	
*TLR3*	XM_005500210.2	GGAAATCTCTTTGGCCTTG	91
		GAATCGCCGTGTTTGATAA	
*TLR4*	XM_005498384.2	TGTTTACAGCCACCCAGTT	145
		GGACTCAGCCTCATTGCT	
*TLR5*	KM086723.1	CAGCTTGTGTGGGAGAGAGG	103
		CCTGTGGAAGAGCACTGAGG	
*TLR7*	XM_005512700.2	CAATCAGTCCCAAAGCTCA	79
		TAAACATCTGTGCTGGCAA	
*TNF-α*	XM_005506079.2	GTGTTCAGTCCCTCCTCGTT	230
		TCAATCACAAGCAATGGGAGC	
*GAPDH*	NM_001282835.1	CCGGGCTGCTATTCTCTCTG	141
		TTCCCATTCTCGGCCTTGAC	

*CLDN, claudin; GAPDH, glyceraldehyde-3-phosphate dehydrogenase; MUC, mucin; OCLN, occludin; PIGR, polymeric immunoglobulin receptor; TJP, tight junction protein; TLR, toll-like receptor; TNF, tumor necrosis factor*.

### Statistical Analysis

The data obtained from this experiment except 16S rDNA high-throughput sequencing were subjected to an independent-sample t-test in SPSS 24.0 (SPSS Inc., Chicago, IL, United States) for Windows. Plotting was performed with GraphPad Prism 7.0 (GraphPad Software Inc., San Diego, CA, United States). Values were presented as means with their standard errors of eight squabs. The data obtained from 16S rDNA high-throughput sequencing were subjected to Mann–Whitney test. In addition, the effect of early weaning on microbiota compositions was evaluated by Mann–Whitney test with false discovery rate (FDR) correction. The level of significance was chosen at *p* < 0.05. In addition, highly significant differences were considered when the *p* value <0.01. Pearson correlation analysis was performed by PerformanceAnalytics R package (1.60.0). Correlation was considered significant at *p* < 0.05, highly significant at *p* < 0.01, and extremely significant at *p* < 0.001. Correlation Network was performed using the OmicStudio tools at https://www.omicstudio.cn/tool.

## Results

### Growth Performance

**Figure 1:**
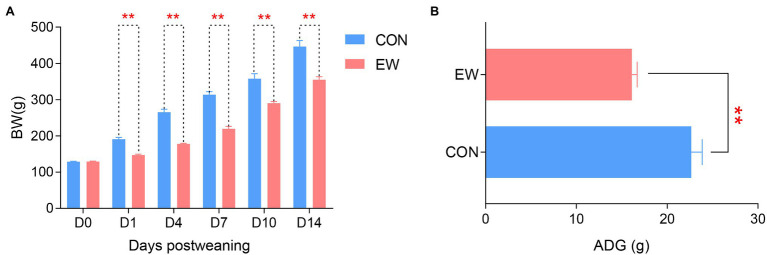
Effects of early weaning on BW **(A)** and ADG **(B)** of squabs. D1, D4, D7, D10, and D14 means 1, 4, 7, 10, and 14 days postweaning, respectively. Values are means with their standard errors of eight squabs. ^**^ mean *p* < 0.01, within each time points postweaning. BW, body weight; ADG, average daily gain; CON, control group; EW, early weaning group.

Figure 1 shows BW and ADG of squabs in both CON and EW groups. Early weaning resulted in a decreased (*p* < 0.01) BW in squabs at each time point (D1, D4, D7, D10, and D14 postweaning). The ADG in the EW group decreased (*p* < 0.01) significantly compared with that in the CON group.

### Microbiota Analyses

**Figure 2:**
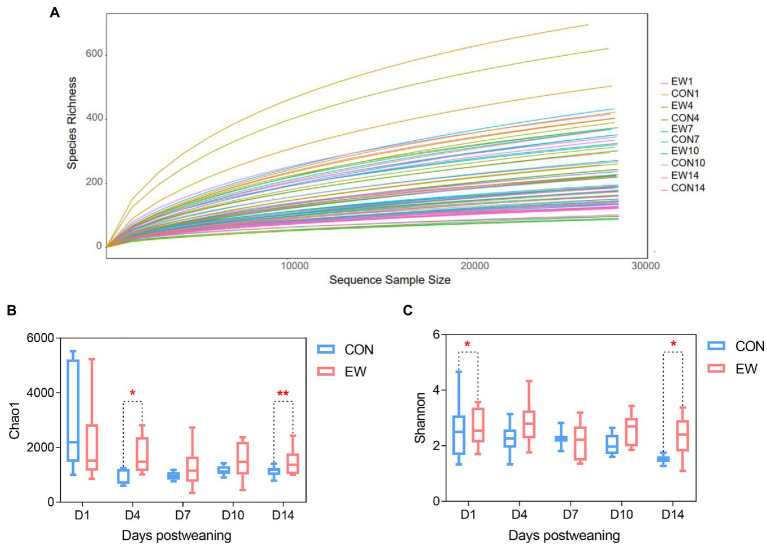
Effects of early weaning on α-diversity of ileal microbiota in squabs. D1, D4, D7, D10, and D14 means 1, 4, 7, 10, and 14 days postweaning, respectively. **(A)** Rarefaction curves; **(B)** richness index: Chao 1; **(C)** diversity index: Shannon. Values are means with their standard errors of eight squabs. ^*^ and ^**^ mean *p* < 0.05 and *p* < 0.01, respectively, within each time points postweaning. CON, control group; EW, early weaning group.

**Figure 3:**
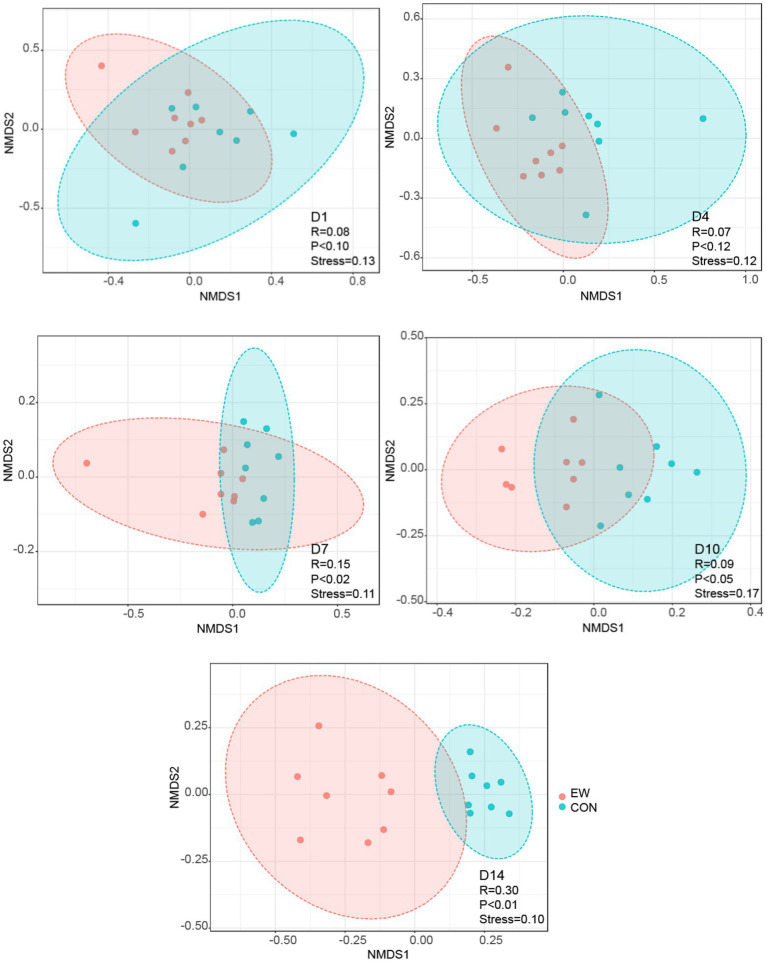
Nonmetric multidimensional scaling (NMDS) analysis (Bray–Curtis distance) comparing changes in ileal bacterial community between the control (CON) and the early weaning (EW) groups. D1, D4, D7, D10, and D14 means 1, 4, 7, 10, and 14 days postweaning, respectively.

A total number of 7,517,099 high-quality reads, with an average of 93,964 V3-V4 16S rRNA gene sequence reads were obtained for each sample. The length of the sequence reads after primer removal ranged between 407 and 429 bp. The average number of OTUs detected by the analysis was 631 (rang from 164 to 2,827), based on 97% nucleotide sequence identity between reads. The rarefaction curves (Figure 2A) leveled off as number of sequences increased revealing that there was sufficient OTU coverage to accurately describe the bacterial composition of each group. Comparing to the control group, the ileal microbiota in early weaned squabs had greater richness based on the Chao1 index at D4 (*p* < 0.05) and D14 (*p* < 0.01) postweaning and had more diverse (*p* < 0.05) based on the Shannon index at D1 and D14 postweaning (Figures 2B,C). As shown in the NMDS figure (Figure 3), the plots for different groups were distinctly separated from D7 postweaning (*p* < 0.05). Pairwise comparison between CON and EW groups showed that D14 postweaning presented a significantly different bacterial community (*p* < 0.01).

**Figure 4:**
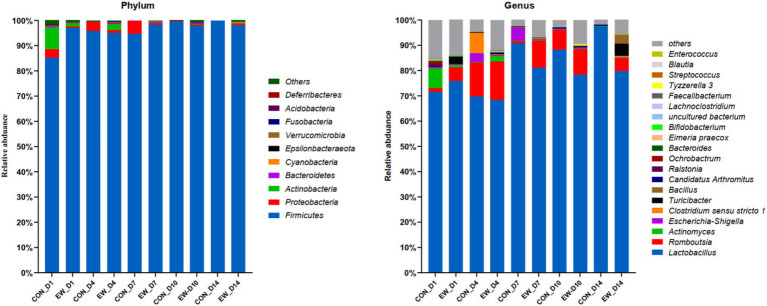
Relative abundance of ileal microbial community at the phylum and genus levels in squabs in CON group and EW weaning group. D1, D4, D7, D10, and D14 means 1, 4, 7, 10, and 14 days postweaning, respectively.

**Figure 5:**
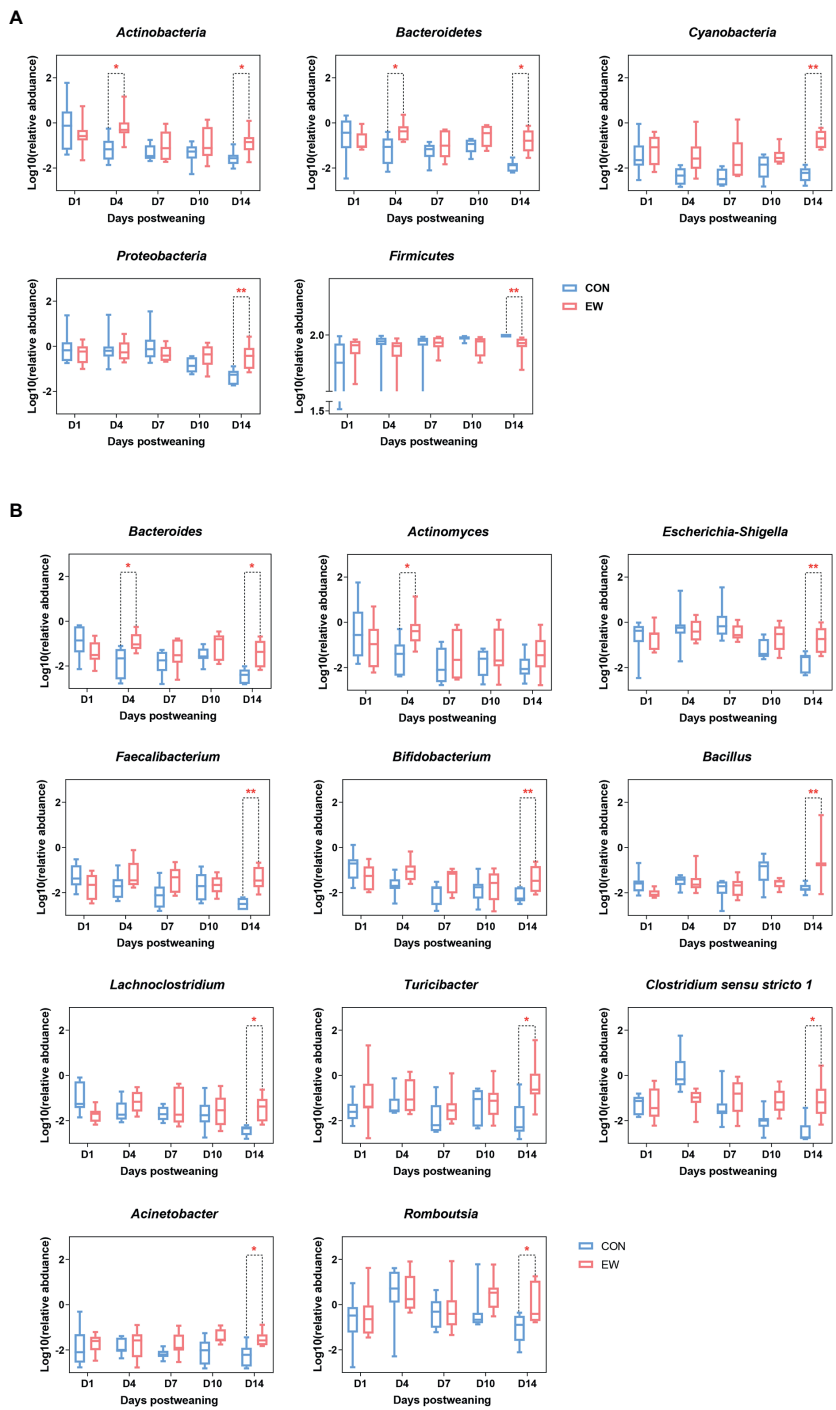
Significantly altered relative abundance of ileal microbial community at the phylum and genus levels in squabs. **(A)** Significantly altered phyla. **(B)** Significantly altered genera. D1, D4, D7, D10, and D14 means 1, 4, 7, 10, and 14 days postweaning, respectively. Values are means with their standard errors of eight squabs. ^*^ and ^**^ mean *p* < 0.05 and *p* < 0.01, respectively, within each time points postweaning. CON, control group; EW, early weaning group.

Relative abundances of microbiota compositions with respect to the levels of phylum and genus are shown in Figure 4 and Additional File 1. Among them, *Firmicutes* was identified as the dominant phylum across all groups, and *Lactobacillus* was the dominant genus in all groups. Early weaning significantly increased the relative abundance of *Actinobacteria* (*p* < 0.05), *Bacteroidetes* (*p* < 0.05), *Cyanobacteria* (*p* < 0.01), and *Proteobacteria* (*p* < 0.01), and decreased the relative abundance of *Firmicutes* (*p* < 0.01) at D4 or D14 postweaning (Figure 5A). Early weaning significantly increased the relative abundance of *Bacteroides* (*p* < 0.05) at both D4 and D14 postweaning, increased the relative abundance of *Actinomyces* (*p* < 0.05) at D4 postweaning, and increased the relative abundance of *Escherichia-Shigella* (*p* < 0.01), *Faecalibacterium* (*p* < 0.01), *Bifidobacterium* (*p* < 0.01), *Bacillus* (*p* < 0.01), *Lachnoclostridium* (*p* < 0.05), *Turicibacter* (*p* < 0.05), *Clostridium sensu stricto 1* (*p* < 0.05), *Acinetobacter* (*p* < 0.05), and *Romboutsia* (*p* < 0.05) at D14 postweaning (Figure 5B).

### Analysis of Intestinal Mucins and Immunoglobulins

**Figure 6:**
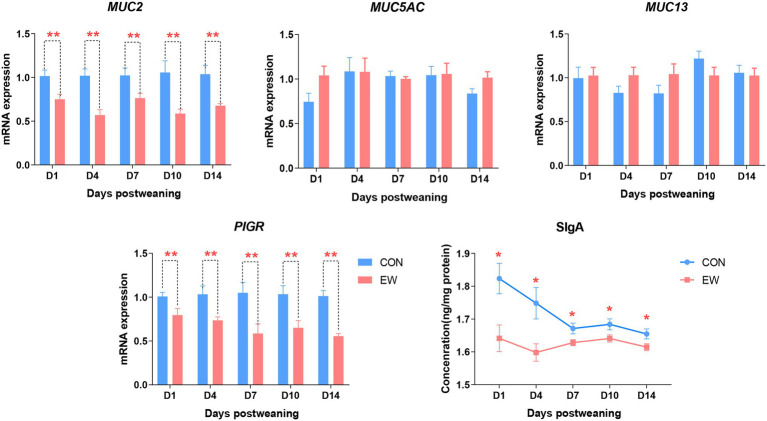
Effects of early weaning on mRNA expression of mucins (*MUC*), polymeric immunoglobulin receptor (*PIGR*), and concentration of secretory IgA (SIgA) in the ileum of squabs. D1, D4, D7, D10, and D14 means 1, 4, 7, 10, and 14 days postweaning, respectively. Values are means with their standard errors of eight squabs. ^*^ and ^**^ mean *p* < 0.05 and *p* < 0.01, respectively, within each time points postweaning. CON, control group; EW, early weaning group.

Figure 6 shows the mRNA expression of genes encoding polymeric immunoglobulin receptor (PIGR) and mucins, and the concentration of SIgA in the both CON and EW groups at different time points. Comparing to the control group, the mRNA levels of *PIGR* (*p* < 0.01) and mucin-2 (MUC2; *p* < 0.01) and the concentration levels of SIgA (*p* < 0.05) decreased significantly at each time point postweaning in the ileum from EW group. There were no significant differences in mucin-5 AC (MUC5AC) and mucin-13 (MUC13) mRNA expression levels between CON and EW groups.

### Intestinal Morphometric Traits

**Figure 7:**
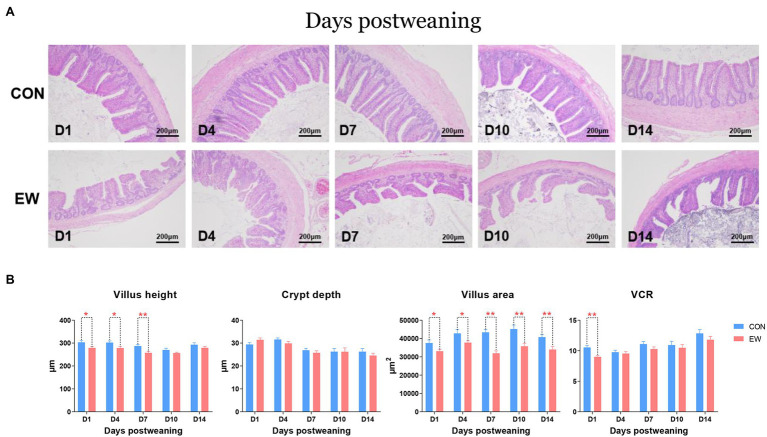
Representative haematoxylin and eosin-stained morphology **(A)** and morphometric traits **(B)** in the ileum of squabs between the CON and the EW groups. D1, D4, D7, D10, and D14 means 1, 4, 7, 10, and 14 days postweaning, respectively. Values are means with their standard errors of eight squabs. ^*^ and ^**^ mean *p* < 0.05 and *p* < 0.01, respectively, within each time points postweaning. CON, control group; EW, early weaning group; VCR, the ratio of villi to crypt.

Ileal morphometric traits in the both CON and EW groups at different time points are shown in Figures 7A,B. Villus height in the EW group were lower than those in the CON group on D1 (*p* < 0.05), D4 (*p* < 0.05), and D7 (*p* < 0.01) postweaning. There were no significant effects of early weaning on crypt depth, however, a significant decrease (*p* < 0.01) in VCR were observed on D1 postweaning in the EW group. Although villus height in the EW group returned to CON values from D10 postweaning, villus surface area decreased (*p* < 0.01 or 0.05) significantly at each time point postweaning in the ileum.

### Intestinal Mucosal Permeability

The intestinal mucosal permeability of squabs was reflected by the levels of endotoxin, DAO, and D-lactate measured in serum (Table 2). The early weaning resulted in increased (*p* < 0.01or 0.05) endotoxin and DAO levels at one or more time points. There was no significant difference in D-lactate level between CON and EW groups.

**Table 2:** Effects of early weaning on intestinal permeability of squabs.

Items	Group	Days postweaning				
		D1	D4	D7	D10	D14
Endotoxin (EU/mL)	CON	83.82 ± 1.68	87.04 ± 1.83	92.79 ± 2.76	92.11 ± 1.97	83.73 ± 1.58
	EW	101.81 ± 1.77	106.17 ± 1.16	105.12 ± 3.09	100.51 ± 3.23	96.18 ± 1.74
	*p*-value	<0.001	<0.001	0.010	0.045	<0.001
Diamine oxidase (ng/mL)	CON	17.88 ± 0.43	17.82 ± 0.32	20.91 ± 0.36	20.20 ± 0.63	19.34 ± 0.42
	EW	18.57 ± 0.36	20.66 ± 0.46	20.15 ± 0.71	19.54 ± 0.38	19.04 ± 0.58
	*p*-value	0.205	<0.001	0.361	0.390	0.683
D-lactate (nmol/L)	CON	105.13 ± 7.66	106.64 ± 12.95	105.13 ± 9.16	114.50 ± 12.32	114.84 ± 7.26
	EW	97.17 ± 8.74	119.05 ± 6.77	122.00 ± 4.93	116.32 ± 5.76	109.43 ± 4.90
	*p*-value	0.505	0.410	0.127	0.895	0.547

*Values are means with their standard errors of eight squabs*.

*D1, D4, D7, D10, and D14 means 1, 4, 7, 10, and 14 days postweaning, respectively*.

*CON, control group; EW, early weaning group*.

### Analysis of Intestinal Tight Junction Proteins

**Figure 8:**
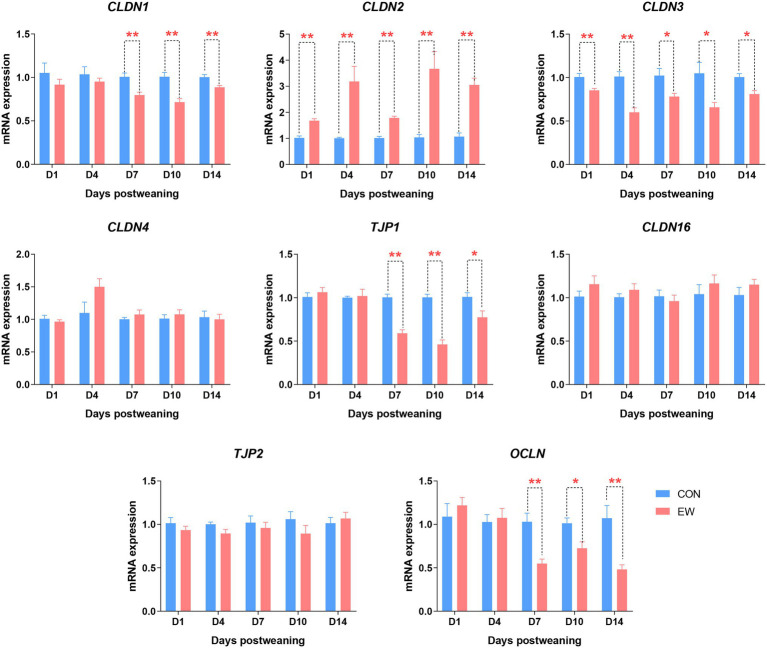
Effects of early weaning on mRNA expression of tight junction proteins in the ileum of squabs. D1, D4, D7, D10, and D14 means 1, 4, 7, 10, and 14 days postweaning, respectively. Values are means with their standard errors of 8 squabs. ^*^ and ^**^ mean *p* < 0.05 and *p* < 0.01, respectively, within each time points postweaning. CON, control group; EW, early weaning group; CLDN, claudin; OCLN, occludin; TJP, tight junction protein.

Figure 8 shows the mRNA expression of genes encoding tight junction proteins in the ileum of both CON and EW groups at different time points. Claudin 1 (*CLDN1*), tight junction protein 1 (*TJP1*), and occludin (*OCLN*) mRNA levels in the EW group were lower (*p* < 0.01 or 0.05) than those in the CON group from D7 to D14 postweaning. Compared to the control, the Claudin 2 (*CLDN2*) mRNA expression level was significantly increased (*p* < 0.01) at all time points postweaning. On the other hand, the Claudin 3 (*CLDN3*) mRNA expression level significantly decreased (*p* < 0.01 or 0.05) at all-time points postweaning. There were no significant differences in Claudin 4 (*CLDN4*), Claudin 16 (*CLDN16*), and tight junction protein 1 (*TJP2*) mRNA expression levels between CON and EW groups.

### Analysis of Intestinal Toll-Like Receptors

**Figure 9:**
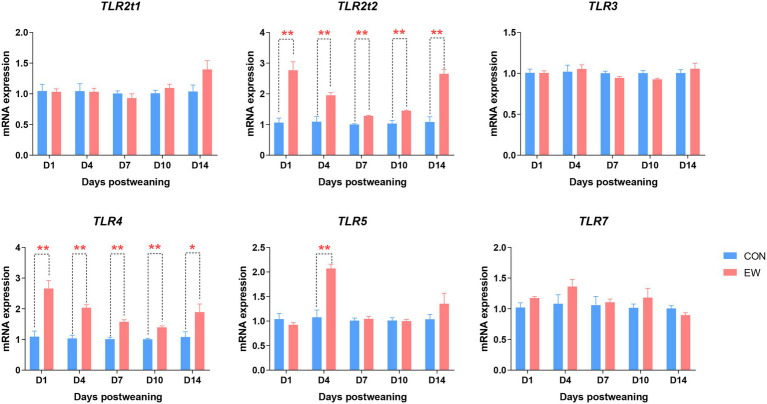
Effects of early weaning on mRNA expression of toll-like receptors (TLR) in the ileum of squabs. D1, D4, D7, D10, and D14 means 1, 4, 7, 10, and 14 days postweaning, respectively. Values are means with their standard errors of eight squabs. ^*^ and ^**^ mean *p* < 0.05 and *p* < 0.01, respectively, within each time points postweaning. CON, control group; EW, early weaning group.

The effect of early weaning on the mRNA expression of genes encoding toll-like receptors (TLRs) during the 14 days after weaning is presented in Figure 9. Compared to the control, the *TLR2t2* and *TLR4* mRNA expression levels were significantly increased (*p* < 0.01–0.05) at all time points postweaning. In the EW group, *TLR5* mRNA level was higher (*p* < 0.01) than that in the CON group on D4 postweaning. There were no significant differences in *TLR2t1*, *TLR3,* and *TLR7* mRNA expression levels between CON and EW groups.

### Analysis of Intestinal Cytokines

**Figure 10:**
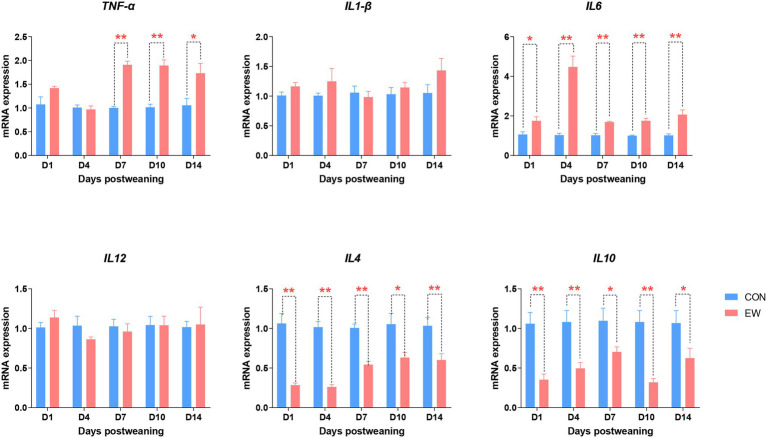
Effects of early weaning on mRNA expression of cytokines in the ileum of squabs. D1, D4, D7, D10, and D14 means 1, 4, 7, 10, and 14 days postweaning, respectively. Values are means with their standard errors of eight squabs. ^*^ and ^**^ mean *p* < 0.05 and *p* < 0.01, respectively, within each time points postweaning. CON, control group; EW, early weaning group; TNF, tumor necrosis factor.

The effects of early weaning on proinflammatory and anti-inflammatory cytokine mRNA levels during the 14 days after weaning is presented in Figure 10. Tumor necrosis factor-α (*TNF-α*) mRNA levels in the EW group were higher (*p* < 0.01 or 0.05) than those in the CON group from D7 to D14 postweaning. Compared to the control, the *IL6* mRNA expression level was significantly increased (*p* < 0.01 or 0.05) at all time points postweaning. On the other hand, the *IL4* and *IL10* mRNA expression levels were significantly decreased (*p* < 0.01 or 0.05) at all time points postweaning. There were no significant differences in *IL1-β* and *IL12* mRNA expression levels between CON and EW groups.

### Gene Expression Correlation Analysis

**Figure 11:**
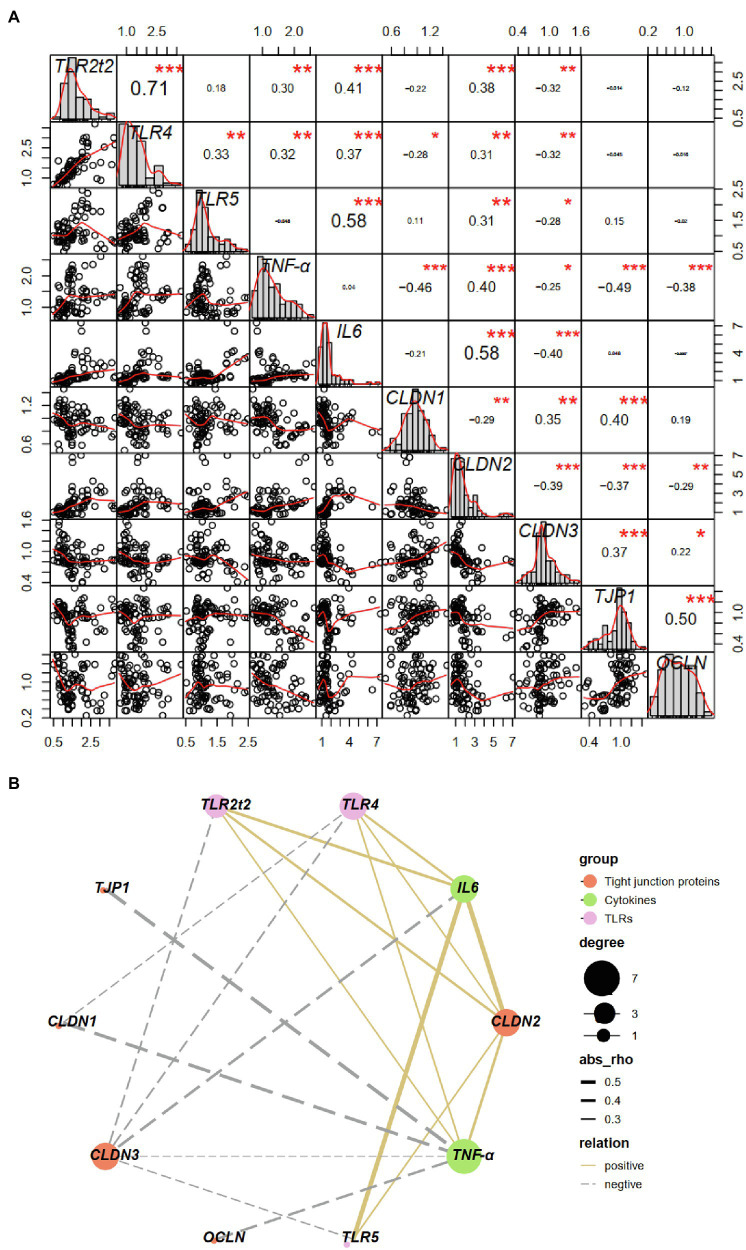
Correlation between toll-like receptors and proinflammatory cytokines and tight junction proteins based on Pearson correlation analysis **(A)** and correlation network analysis **(B)**. ^∗^*p* < 0.05, ^∗∗^*p* < 0.01, ^∗∗∗^*p* < 0.001. CLDN, claudin; OCLN, occludin; TJP, tight junction protein; TLRs, toll-like receptors; TNF, tumor necrosis factor.

A Pearson’s correlation analysis was carried out to evaluate the potential link between TLRs and proinflammatory cytokines and tight junction proteins (Figures 11A,B). There was a significant correlation (*p* < 0.01 or 0.05) between the expression of *TLR2t2*/*TLR4*/*TLR5* and *CLDN2*/*CLDN3*, and both *TLR2t2*/*TLR4*/*TLR5* and *CLDN2* levels showed a significant positive correlation (*p* < 0.01) with *IL6* level, whereas *CLDN3* level showed a significant negative correlation (*p* < 0.01) with IL6 level. Besides, both *TLR2t2*/*TLR4* and *CLDN2* levels showed a significant positive correlation (*p* < 0.01 or 0.05) with *TNF-α* level, whereas *CLDN3* level showed a significant negative correlation (*p* < 0.01) with TNF-α level. The expression of *TLR4*, *TNF-α*, and *CLDN1* showed a significant positive correlation (*p* < 0.01 or 0.05) between each other. Finally, the expression of *TNF-α* was negatively correlated (*p* < 0.01) with that of *TJP1* and *OCLN*.

## Discussion

Our data demonstrated that weaning induced acute and sequential decline of growth performance in squabs during the first 14 days postweaning. Just as mammals, weaning of squabs is associated with social, environmental and dietary stress. Multiple lines of evidence derived from research studies on porcine and ruminant show the early life stress induces immediate and long-term deleterious effects on intestinal functions including intestinal barrier dysfunction, disturbed intestinal microbiota, and dysregulated intestinal immune activation (Smith et al., 2010; Li et al., 2018). It is well established that the maintenance of normal intestinal functions is important for animal health and hence productivity. As such, the impaired intestinal functions accompanied with abrupt weaning seems to be the main reason for the poor growth rate after weaning. However, understanding of weaning stress on intestinal functions is very limited in altricial birds, especially in squabs. Thus, the effects of early weaning on intestinal microbiota and barrier function were further measured in this study.

The intestinal microbiota contributes to the maintenance of host barrier function and performance (Yoon et al., 2014). Consistent with previous studies on mammals (Chen et al., 2017; Li et al., 2018) and precocial birds (Yu et al., 2019), this study demonstrated that *Firmicutes* was the most dominant phyla in the ileal microbiota in both CON and EW groups during the whole investigated period. At genus level, *Lactobacillus* is characterized as the most dominant genus in all groups. Our results correspond to a recent study by Yu et al. (2019) who found the genus *Lactobacillus* displayed the highest relative average abundance from days 14 to 42 in chickens. Although the dominant phyla and genus was the same for both CON and EW groups in our study, each group had a distinct ileal microbiota composition as reflected by the alpha diversity indices, the clustering of samples by group in NMDS, and by the relative abundance of microbes at both phylum and genus levels. Many studies have shown that weaning stress can affect the intestinal microbiota of young animals (Meale et al., 2016; Chen et al., 2017). In the current study, ileal microbiota of early weaning squabs had a greater richness and was more diverse than that of controls during postweaning period; this finding is congruent with previous studies in mammals (Chen et al., 2017; Li et al., 2018). Mounting evidence suggests that the composition and diversity of gut microbes are influenced by diet composition and environment (Liu et al., 2017), thus, it is reasonable to speculate that the significantly increased bacterial diversity during the postweaning period may be partly due to the diet transition from pigeon milk to artificial feed. Furthermore, a high diversity of intestinal microbiota is theoretically considered to provide ‘functional redundancy’ that helps an ecosystem maintain its resilience and stability after environmental stresses (Konopka, 2009). Our abundance results showed that early weaning significantly changed the relative abundances of five phyla (*Actinobacteria*, *Bacteroidetes*, *Cyanobacteria*, *Proteobacteria*, and *Firmicutes*) and 11 genera (*Bacteroides*, *Actinomyces*, *Escherichia-Shigella*, *Faecalibacterium*, *Bifidobacterium*, *Bacillus*, *Lachnoclostridium*, *Turicibacter*, *Clostridium sensu stricto 1*, *Acinetobacter*, and *Romboutsia*). Most of these microbes’ abundances also altered in other biological intestines during postweaning period, but they have diverse shifts (Abecia et al., 2017; Chen et al., 2017; Li et al., 2018). The reason of the distinct shifts could be that most of these microbes may perform different physiological functions in different host organisms (Yu et al., 2019). It is important to note that immature gut microbiota is sensitive to early life stress and vulnerable to be disturbed. Previous study in our lab showed that a presumably more stable microbiota is not yet established by day 21 of age in squabs (Xu et al., 2020). Taken together, our results based on alpha diversity indices and abundance analyses suggested that early weaning disturbed the intestinal microbiota of squabs which could lead to intestinal distress. Another interesting finding of this study is that the intestinal microbiota was very different between CON and EW groups on D14 postweaning. It is speculated that differences in microflora were mainly due to the influence of early microbial colonization.

Intestinal secretory mucins and immunoglobulins have long been considered to be the first protective barrier against adhesion and invasion of pathogenic microorganisms to the mucosa (Deplancke and Gaskins, 2001; Macpherson et al., 2008). Our current results found that the mRNA expression of *MUC2* encoding mucin glycoproteins was down-regulated in weaning squabs; this finding is in line with a previous study on piglets (Yang et al., 2016). Mucin glycoproteins are considered to enhancing the mucosa immune system by the accumulation of SIgA (Xun et al., 2018). As expected, the mRNA expression of *PIGR,* which is responsible for SIgA transporting from the lamina propria layer to the intestinal mucosa, and the concentration of SIgA decreased due to early weaning stress in this study. In contrast to this finding, Xun et al. (2018) reported that early weaning significantly increased intestinal SIgA content in piglets. Because SIgA also plays an important role in the selection and maintenance of a spatially diversified intestinal bacterial community (Suzuki et al., 2007), we speculated that the decreased SIgA could be a compromise to the increased richness of ileal microbiota in squabs. Nevertheless, our results suggested that weaning destructed the protective coat on the surface of intestine, and this destruct perform may be attributed to the disturbance of intestinal microbiota.

The integrity of intestinal architecture is also crucial for the maintenance of barrier functions. As already reported in mammals, weaning was followed by significant morphological changes in the gut, i.e., a reduction in villus height and crypt depth, as well as a reduced villus surface area (Hu et al., 2013; Xiao et al., 2014). The present study showed that the villus height and VCR in EW group were decreased compared with those of control during the earlier postweaning period, but returned to the control value during the later postweaning period. Similarly, Hu et al. (2013) reported that villus height and crypt depth returned to the preweaning value at 14 days postweaning in the porcine jejunum. However, our data revealed that early weaning displayed a sustained decrease in villus surface area during the 14 days postweaning period. It has been proven that these morphological changes are associated with intestinal microbiota composition (Gancarcikova et al., 2009). Our results indicate that early weaning has a lasting effect rather than a transient effect on intestinal architecture in pigeons that coincide with changes to the microbiota. Moreover, the reduction of villus surface area postweaning implicates a reduced nutrients absorption as well.

The integrity of the intestinal barrier was fundamental to maintain the epithelial permeability by preventing the entry of enteric pathogens and endotoxin. An increase in the intestinal permeability has been shown to correlate closely with villus atrophy (Jeurissen et al., 2002). In the present experiment, serum levels of endotoxin, DAO, and D-lactate, which are negatively related to the intestinal permeability, were used to reflect the extent of damage of intestinal mucosa. We found that endotoxin and DAO levels were significantly increased at one or more time points postweaning. Moreover, the endotoxin was still at a higher level at D14 postweaning in EW group, indicating that early weaning resulted in lasting increase of intestinal permeability in squabs. Intestinal permeability is primarily regulated by a well-organized system referred to as the tight junction. To better clarify the molecular mechanism for the impaired intestinal barrier integrity in weaning squabs, we determined the changes in the mRNA expression of tight junction proteins, such as claudins, occludin, and zonula occludens. Our results showed that early weaning significantly decreased the mRNA expression of *CLDN1*, *CLDN3*, *OCLN*, and *TJP1* (also known as *ZO-1*), but increased the mRNA expression of *CLDN2* from D1 or D7 to D14 postweaning. *CLDN1*, *CLDN3*, *OCLN*, and *TJP1* are known to be “tightening” tight junction proteins, whereas *CLDN2* expression is associated with increased intestinal permeability and decreased tightness of the epithelial barrier (Suzuki et al., 2011; Ariyadi et al., 2013). These results coincided with the increased intestinal permeability in weaned squabs, which may indicate sustained damage of intestinal barrier integrity. Similar results were also found in mammals, in which early weaning resulted in sustaining dysfunction of the intestinal barrier based on altered expression of tight junction proteins and increased mucosal permeability (Hu et al., 2013; Xiao et al., 2014). It is now well established that claudins are important in establishing the tight junction pore pathway, while occludin and zonula occludens are important in the leak pathway. The novel and significant result of the present study is that the effect of early weaning on leak pathway permeability is not immediate in squabs. Rather it was most apparent in the second week postweaning. Although the exact mechanism involved remains unclear, our findings can provide new insight into regulation of intestinal tight junction in animals under early life stress.

Due to the decreased expression of mucins and SIgA and the increased intestinal permeability, penetration of bacteria across the mucosa may be enhanced within the first 14 days postweaning in squabs. Translocated bacteria can be recognized by TLRs, which can detect a wide variety of pathogen-associated molecular patterns (PAMPs) from bacteria. Our data showed that the expression of *TLR2t2* and *TLR4* could be rapidly activated to recognize the bacteria PAMPs in the small intestine of newly weaned squabs, while the expression of *TLR5* could only be activated on D4 postweaning. Moreover, the activation effect of *TLR2t2* and *TLR4* caused by weaning stress were observed throughout the 14 days postweaning. These results indicate that *TLR2t2* and *TLR4* have potentially more important roles than other TLR genes in responding to the microbial translocation and modulating the host innate immune response during postweaning development period. In agreement with our findings, Li et al. (2018) and Tao et al. (2015) reported that weaning stress allowed more bacterial products to breach the mucosal barrier to stimulate the expression of *TLR2* and *TLR4* genes.

The increased expression of TLR genes in the early weaning squabs suggests that weaning stress activates immune cell responses in the intestine. As such, we further analyzed the effect of weaning stress on the expression of genes encoding cytokines during the 14 days after weaning. Once pathogens have breached the mucosal barriers of the intestinal tract, engagement of TLRs on mucosal cells and consequent activation of signaling cascades including Nuclear factor-κB (NF-κB), activator protein 1, interferon response factors and mitogen-activated protein kinases can promote the production of proinflammatory cytokines, such as TNF-α, IL1-β, IL6, and IL12 (Lavelle et al., 2010). Many studies have shown that early weaning was associated with the alternations in the expression of proinflammatory cytokines in the intestine of mammals (Hu et al., 2013; Mclamb et al., 2013). In our study, early weaning significantly increased the ileal mRNA levels of *TNF-α* from D7 to D14 postweaning, and *IL6* during the whole 14 days post weaning, but had no effect on the expression of *IL1-β* and *IL12*. These results suggest that the intestinal mucosal immune system becomes activated under the stress of early weaning and that inflammation occurred mainly attribute to the upregulation of *TNF-α* and *IL6*. Moreover, a decreased expression of *IL-4* and *IL10* was observed in response to early weaning stress in the current study. IL4 and IL10, as anti-inflammatory cytokines, are important for the attenuation of the inflammatory process by inhibiting the production of proinflammatory cytokines. In contrast, Hu et al. (2013) demonstrated that no significant alternations of anti-inflammatory cytokines mRNA were observed during the first 2 weeks after weaning in piglets. The decreased expression of anti-inflammatory cytokines in our study is most likely due to the synergistic effects of cytokines on the inflammatory processes; that is, higher levels of proinflammatory cytokines may inhibit the production of anti-inflammatory cytokines (Gao et al., 2013).

Overproduction of proinflammatory cytokines has a negative regulation on intestinal barrier integrity in mammals (Bruewer et al., 2003; Al-Sadi, 2009). For instance, TNF-α and IL6 induced by the activation of TLR4 play a crucial role in regulation of intestinal tight junction proteins (Bruewer et al., 2003). However, few data are available regarding the relationship between the proinflammatory cytokines and intestinal barrier function in squabs under early weaning stress. To better clarify the molecular mechanism for the impaired mucosal barrier function in weaning squabs, we further assessed the correlation between the expression of TLRs and proinflammatory cytokines and tight junction proteins. We found that *TLR2t2*/*TLR4*/*TLR5*-dependent induction of *TNF-α* and/or *IL6* may play a role in regulation of pore pathway related tight junction protein genes (i.e., *CLDN1*, *CLDN2*, and *CLDN3*). In addition, *TNF-α* also may play a role in regulation of leak pathway related tight junction protein genes (i.e., *TJP1* and *OCLN*) in a *TLR*-independent manner. However, considering the vast differences in microbiome composition between the CON and EW groups, we cannot infer specific links between *TLR* expression and bacterial taxa through correlation analysis. Nevertheless, the observed multiple significant correlations between mRNA levels of TLRs and proinflammatory cytokines and tight junction proteins provide some insight into potential host–microbe interactions in the small intestine of squabs under weaning stress. Besides, further experiments regarding the detection of the expression of downstream factors, such as NF-κB/IL6, should be performed to show the TLRs signaling pathway by changing microbiota diversity within the digestive tract in weaned squabs.

To conclude, as in mammals, the present study revealed that the impaired intestinal barrier functions accompanied with early weaning stress seems to be the main reason for the poor growth rate after weaning in pigeon squabs. Overall our data demonstrate that early weaning stress in squabs induces immediate and long-lasting deleterious effects in the intestinal functions of squabs. Furthermore, the disturbance of intestinal microbiota consequence of early weaning stress in squabs coincided with dysfunction of intestinal mucosal barrier and activation of inflammation cell responses that were possibly mediated *via* the activation of TLRs. However, it is important to note that gene expression does not necessarily correlate with the protein expression; thus, studies on protein expression are necessary in the future to gain a clear understanding of the effects of early weaning on mucosal barrier functions in squabs.

## Data Availability Statement

The original contributions presented in the study are included in the article/Supplementary Material, further inquiries can be directed to the corresponding author.

## Ethics Statement

The animal study was reviewed and approved by the Animal Care and Welfare Committee of Animal Science College and the Scientific Ethical Committee of Zhejiang University.

## Author Contributions

QX: conceptualization, methodology, data curation, and writing—original draft preparation. WZ: sample collection and software. JL: visualization and investigation. HJ: writing—review and editing. XZ: supervision, writing—review and editing. XD: project administration and funding acquisition. All authors contributed to the article and approved the submitted version.

## Funding

This work was supported by the National Natural Science Foundation of China (31902173) and Zhejiang Provincial Natural Science Foundation (LY22C170002).

## Conflict of Interest

The authors declare that the research was conducted in the absence of any commercial or financial relationships that could be construed as a potential conflict of interest.

## Publisher’s Note

All claims expressed in this article are solely those of the authors and do not necessarily represent those of their affiliated organizations, or those of the publisher, the editors and the reviewers. Any product that may be evaluated in this article, or claim that may be made by its manufacturer, is not guaranteed or endorsed by the publisher.

## Abbreviations

ADG: Average daily gain
BW: Body weight
CLDN: Claudin
CON: Control group
DAO: Diamine oxidase
EW: Early weaning group
GAPDH: Glyceraldehyde-3-phosphate dehydrogenase
MUC: Mucin
NF-κB: Nuclear factor-κB
NMDS: Nonmetric multidimensional scaling
PIGR: Polymeric immunoglobulin receptor
OCLN: Occludin
SIgA: Secretory IgA
TJP: Tight junction protein
TLRs: Toll-like receptors
TNF: Tumor necrosis factor
